# Mapping Scientific Research on High-Intensity Interval Training (HIIT) in Overweight Populations (2011–2024)

**DOI:** 10.3390/sports14010038

**Published:** 2026-01-14

**Authors:** Juan David Paucar-Uribe, Andrés Julián Rendón-Sanchéz, Mauricio Vladimir Peña-Giraldo, Kevin Ricardo Forero González, Anyi Tatiana Sanabria Moreno, Boryi A. Becerra-Patiño, Laura del Pilar Prieto Mondragon, Jorge Olivares-Arancibia, Rodrigo Yáñez-Sepúlveda, José Francisco López-Gil

**Affiliations:** 1 Faculty of Physical Education, National Pedagogical University, Bogotá 480100, Colombia; jdpaucaru@upn.edu.co (J.D.P.-U.); ajrendons@upn.edu.co (A.J.R.-S.); mvpenag@upn.edu.co (M.V.P.-G.); kforerog@upn.edu.co (K.R.F.G.); atsanabriam@upn.edu.co (A.T.S.M.); babecerrap@pedagogica.edu.co (B.A.B.-P.); 2 Faculty of Health Sciences, Universidad de Ciencias Aplicadas y Ambientales, Bogotá 480100, Colombia; laprieto@udca.edu.co; 3 AFySE Group, Research in Physical Activity and School Health, School of Physical Education, Faculty of Education, Universidad de las Américas, Santiago 7500975, Chile; jorge.olivares.ar@gmail.com; 4 Faculty Education and Social Sciences, Universidad Andrés Bello, Viña del Mar 2520000, Chile; rodrigo.yanez.s@unab.cl; 5 School of Medicine, Universidad Espíritu Santo, Samborondón 092301, Ecuador; 6 Faculty of Health Sciences, Universidad Autónoma de Chile, Santiago 7500000, Chile

**Keywords:** lifestyle, cardiorespiratory fitness, nutrition, risk factors, prevention

## Abstract

**Background**: Several studies have investigated the importance of physical exercise (PE) in overweight and obese populations; however, to date, no bibliometric study has analyzed research trends in high-intensity interval training (HIIT) in overweight and obese populations across the entire life course. **Objective**: To analyze the scientific output of HIIT trends in overweight and obese populations. **Method**: Theoretical study using bibliometrics as a research technique. A total of 282 studies were identified in the Web of Science and PubMed databases for analysis with VOSViewer software 1.6.20. The equation used was (“High-intensity interval training” OR HIIT) AND (overweight OR obesity OR “risk factors” OR “obesity risk”). **Results**: The year 2022 was the most productive year (*n* = 46). Most of the documents are research articles (81%), followed by review articles (15%). Most studies do not specify the characteristics of the sample, only mentioning the application of a HIIT program in overweight or obese individuals in (65.6%) of the total articles found. There is low scientific output in research focused on women (23.4%). The most frequently occurring words were “exercise” (*n* = 145), “obesity” (*n* = 131), “high-intensity interval training” (*n* = 81), “overweight” (*n* = 78), “physical activity” (*n* = 73), “body composition” (*n* = 46), “weight loss” (*n* = 45), “health” (*n* = 42), and “cardiorespiratory fitness” (*n* = 40). **Conclusions**: Scientific research has advanced the understanding of the impact of HIIT in relation to excess weight, with total fat reduction being one of the most frequently reported variables and no differences observed between sexes. HIIT has shown benefits in populations with overweight and obesity when compared with low-intensity training programs.

## 1. Introduction

Cardiovascular disease (CVD) is the leading cause of death worldwide [1]. It is a preventable disease, so it is necessary to develop preventive measures that help encourage healthy lifestyle habits such as regular exercise, adequate sleep, smoking cessation, healthy eating habits, and physical activity (PA) [2,3].

Obesity and overweight have become among the most dangerous diseases in the last century [4], with pediatric obesity being the first indicator that can lead to morbidity and mortality risks in adults [5]. In countries such as China, Russia, and South Africa, obesity has been shown to be related to diseases such as hypertension, stable and unstable angina, and insulin resistance [6], with obesity being a precursor to comorbidities, cardiovascular diseases, and certain types of cancer [7]. Obesity can be defined as the excessive accumulation of fat in the body with an abnormal distribution, with body mass index (BMI, kg/m^2^) being the most widely used indicator in epidemiological studies and clinical practice [8]; however, this method has major limitations in its results, especially for detecting excess body fat [9]. In contrast, there are now more effective methods for analyzing body composition via gold standard tests, such as bone density (DXA) testing [10].

Moreover, excess body mass has reached 2 billion people, representing almost 30% of the entire global population [1], where it is statistically projected that 57.8% of the adult population will suffer from this disease, forcing the health sector to develop actions to reduce obesity rates [11]. However, there are many recommendations that can help improve overweight and obesity. One of them is to improve and modify bone density through lifestyle habits, such as reducing food intake (caloric deficit) and increasing PA (caloric expenditure) [12]. Furthermore, epidemiological research has revealed a relationship between PA and improved cardiorespiratory and muscular health [13].

However, one of the protective factors for preventing CVD [14], metabolic disorders [15], overweight, and obesity [16] is PA, Likewise, PA levels are decisive in the fight against CVD, overweight, and obesity [2,17,18,19]. The World Health Organization (WHO) guidelines recommend between 75 and 150 min of vigorous PA [20], and the implementation of physical activity (PA) has been associated with a reduction in cardiovascular risk and a higher quality of life [21]. For example, high-intensity interval training (HIIT) allows for adaptations related to improved aerobic capacity, maximum oxygen consumption (VO2max), and improved metabolic health in different population groups (athletes, physically active people, sedentary people without any apparent disease or disorder) [22]. On the other hand, compared with moderate-intensity continuous training (MCIT), HIIT helps promote cardiometabolic risk factors in obese children and adolescents and improves systolic blood pressure and cardiorespiratory capacity [23].

The methods that have attracted the most attention are HIIT and MICT [24,25] because of their benefits in combating CVD [26]. However, more recent evidence, based on meta-analysis, indicates that although both programs have benefits, HIIT significantly improves cardiorespiratory fitness, with increases in maximum oxygen consumption (VO2max) ranging from 38% to 79%, which has particularly positive effects on overweight and obese individuals [27]. HIIT is characterized by peaks of maximum activity reaching ≥90% of VO2max [28], including maximum power, with values >75%, which corresponds to supramaximal efforts [29] and considers very low-intensity recovery periods [22]. Its significant impact on sports science and PA has been relevant for coaches and researchers because of the physiological adaptive improvements and health benefits in individuals [30].

Studies that implemented high-intensity interval training methods lasting 12 weeks concluded that there was an improvement in insulin sensitivity and physio-metabolic adaptation in individuals with obesity [23]. HIIT has a positive effect on cardiometabolic risk, physical fitness, systolic blood pressure, and the risk of being overweight [31,32]. These research advances should be put into practice to improve PA interventions and processes in different population groups. Additionally, the growing number of HIIT protocols, together with the accelerated increase in publications, has led to marked differences in how interventions are designed and assessed. This variability complicates comparisons between studies. To our knowledge, no bibliometric study analyzing HIIT research trends in individuals with excess weight has been published in scientific literature. Therefore, the objective of this study was to analyze the HIIT landscape between 2011 and 2024 in populations with excess weight via bibliometric research.

## 2. Materials and Methods

### 2.1. Design

This research was conducted via bibliometric techniques to analyze the scientific output of HIIT in excess weight individuals between 2011 and 2024. Bibliometrics is based on a theoretical study due to its nature [33], since its purpose is mediated by the collection of information with different variables, which can be systematized and analyzed according to academic production during a specific time [34]. Bibliometric studies are characterized by highlighting and analyzing a mapping of knowledge in a specific area, which establishes different variables, such as authors, publishing groups, countries, organizations, keywords, and citations [35,36].

### 2.2. Data Extraction

Between 28, 29, and 30 September 2024, two principal investigators conducted a search for documents via the Web of Science (WoS) database, which is one of the most widely used databases in the field of sports research [37,38]. The PubMed database was also considered because of its relevance to the fields of health, medicine, PA, and sports. The PRISMA methodology [39] was used for data analysis, extracting data from the WoS and PubMed databases via the following mechanism. Initially, the following terms were considered (“High-Intensity Interval Training” [Title/Abstract]) OR (“HIIT” [Title/Abstract]) AND (Overweight [Title/Abstract]) OR (obesity) [Title/Abstract]) OR (“risk factors” [Title/Abstract]), which allowed us to identify the best terms for the research topic. Second, the following search equation was used: (“High-Intensity Interval Training” OR HIIT) AND (Overweight OR obesity OR “obesity risk”) with the aim of identifying documents through their title and abstract and establishing the documents most closely related to the study topic (Table 1). The searches were conducted to identify studies without other restrictions in terms of language or study design. Similarly, citation searches were conducted for the key studies included. When it was not possible to obtain the full texts of the articles from institutional subscriptions or open access, attempts were made to contact the corresponding authors directly or search for the documents on ResearchGate.

**Figure 1:**
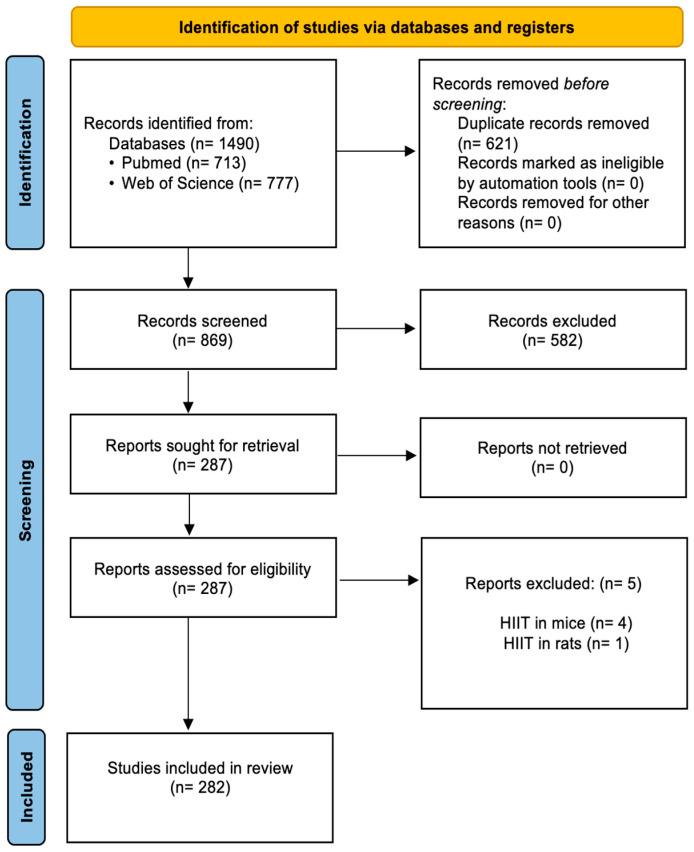
Flow diagram for study selection according to the PRISMA guidelines.

The results found on the WoS platform were 777 documents, and those found on PubMed were 713 documents, for a total of 1490, which were analyzed by two principal investigators. The systematization was carried out at different times for the selection of the documents that made up the study through three stages: identification, screening, and eligibility. During the first phase, the duplication and compatibility of the studies were analyzed against the inclusion criteria, where only 5% of the total studies were significant. At the end of the methodology, 282 articles were selected (Figure 1), which were downloaded in Excel and plain text formats for general mapping.

### 2.3. Eligibility Criteria

The inclusion criteria for studies were as follows: (i) research on interval training in obese individuals; (ii) documents that were peer reviewed by academics; (iii) documents that were freely accessible; (iv) documents without language restrictions; (v) publication date (between 1 January 2011, and 30 September 2024); (vi) the journal was indexed in the Journal Citation Report (JCR). The exclusion criteria were as follows: (i) articles that analyzed animals; (ii) studies that evaluated people with metabolic syndrome that did not specify the disease; (iii) studies that implemented methods such as sprinted interval training and high-intensity functional training.

For the selected documents, a Microsoft Excel spreadsheet was used on the following categories: (1) number of publications per year and number of citations received per year; (2) documents by type; (3) number of articles by population and disease type; (4) authors’ analysis; (5) keyword analysis; (6) citation and country analysis; (7) analysis of the most cited documents; (8) number of citations per article.

### 2.4. Data Analysis

The data were extracted from the WoS and PubMed databases in two different formats: plain text and Excel. This allows descriptive and percentage analyses of the results to be performed via a Microsoft Excel spreadsheet (v. 2006, Microsoft Corporation, Redmond, WA, USA), whereas the data downloaded in plain text format were used for analysis via VOSViewer software (v.6.19., Center for Science and Technology Studies, The Netherlands). This program allows the creation of two-dimensional graphs [40], whose main purpose is to determine the exponential increase in scientific production in certain areas and in consideration of the availability of information found in the databases consulted [41].

The graphs generated via the VOSViewer v.1.6.20. program considered the following categories: occurrence by keywords, citation by documents, citation by journals, citation by countries, citation by authors, cocitation by cited references, cocitation by minimum number of citations of a cited reference, cocitation by number of a cited journal, and cocitation by number of citations of a cited author. A fragmentation analysis was performed with a value of 2 for attraction and 1 for repulsion. In addition, different laws that make up bibliometrics have been used as research techniques [42]. Initially, Price’s law was used using the R2 coefficient [43] to determine the exponential growth of academic productivity in response to a time window. Lotka’s law was used to identify all the authors who have developed the most studies [44] and to determine the most prolific authors [45], while a search and elimination of duplicate coauthors was carried out. Zipf’s Law was used to determine the terms with the highest occurrence [46], and Bradford’s law of scientific dispersion was used to identify the central journals with the most published and cited articles on the subject [47].

## 3. Results

### 3.1. Evolution of the Number of Documents

**Figure 2:**
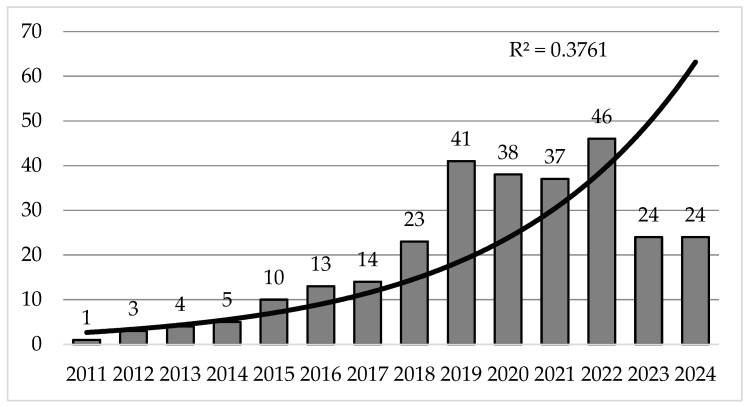
Evolution of the number of annual publications.

An analysis of HIIT in terms of the number of annual publications revealed evidence of linear growth during the first six years from 2011–2017, followed by exponential growth in the next two years (2018–2019). There is subsequently a stabilization phase in terms of article production for a period of three more years, from 2020–2022, with the latter being the most important in terms of production, where it is established with (*n* = 46) and, finally, a significant decrease in the research trend on HIIT, where greater production is projected compared with the previous year (Figure 2).

**Figure 3:**
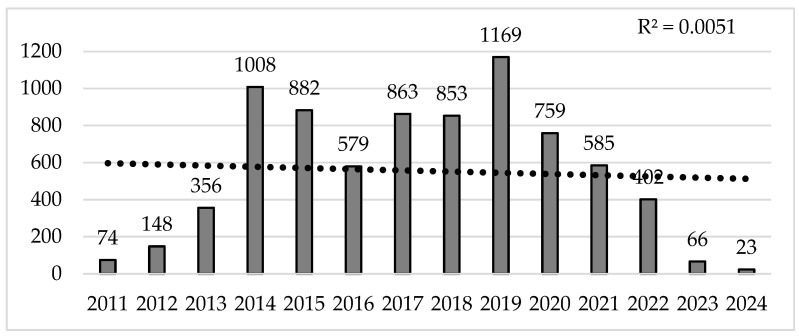
Evolution of the number of citations published per year.

In terms of citations per year, there was an exponential trend in the first three years, from 2011–2014, with 2014 being the second most important year in terms of the number of citations (*n* = 1008), followed by a decline for two consecutive years, 2015 and 2016. Once again, three years later, citations peaked in 2019 (*n* = 1169), followed by a downward trend for five more years (2020–2024), which, together with 2023, were the years with the lowest number of citations in a row (Figure 3).

### 3.2. Documents by Type

In general, we found that most of the documents were research articles, accounting for 81% of the total articles reviewed, followed by review articles, accounting for almost 15% (Table 2).

In the screening of the information, the types of documents were characterized, with empirical articles being the most studied in terms of scientific evidence (82.3%), followed by systematic reviews and meta-analyses (5.7%) (Table 3).

### 3.3. Number of Articles by Population Type and Disease

In terms of population, adults represent the largest 23.8% of the total articles found. However, there is a significant difference between studies focused on females, accounting for 20.6% of the total, and only 13.1% of the studies focused on men. On the other hand, in terms of age groups, there is greater interest in demonstrating the effects of HIIT interventions in adolescents, with 7.8% of the total articles researched, compared with 6.4% corresponding to its effects in children (Table 4).

The highest percentage focused solely on HIIT interventions in overweight or obese individuals, accounting for almost 93% of the articles (Table 5). However, academic research has associated obesity with other types of diseases, such as diabetes type 2 (3.2%), health condition like menopause (2.1%), and cancer (1.1%). There, interest has been shown in demonstrating the effects of a HIIT-type training plan in populations with these types of conditions.

**Figure 4:**
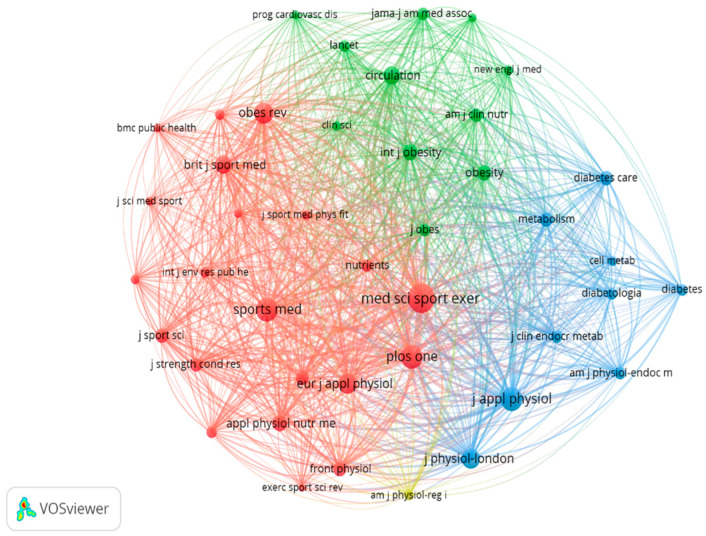
Analysis of cocitations according to journals.

The review considered 282 documents for the analysis of journal cocitations, revealing a total of 2129 journals. To this end, each journal was considered to have received at least 50 citations. Using these criteria, 42 journals were found, among which the following stand out: Medicine and Science in Sport and Exercise with (*n* = 640) citations, PLOS One and Sports Medicine with (*n* = 418) citations, the Journal of Applied Physiology with (*n* = 407) citations, and the Journal Physiology-London with (*n* = 295) citations (Figure 4).

### 3.4. Authors’ Analysis

**Figure 5:**
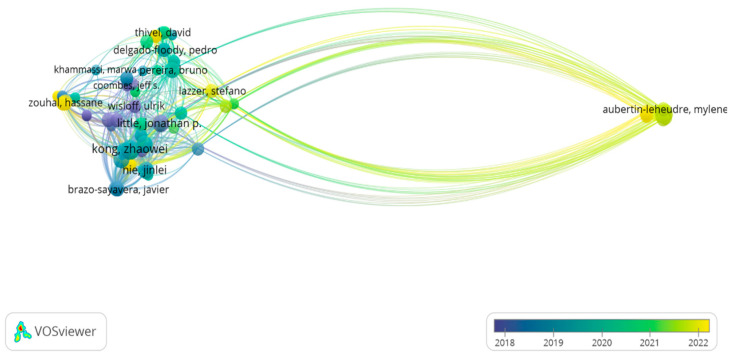
Citation by authors.

For the analysis of authors, each author was considered to have at least three documents; thus, of the 1411 authors identified, only 85 authors met the threshold. The author citation map shows that there are no particularly strong nodes, but over time, production has been led by “Aubertin-Leheudre, M.” between 2021 and 2022, whereas “Kong, Z.” is one of the authors of the main nodes between 2019 and 2020, and “Little, J.” together with “Jung, M.” are the most prominent nodes in 2018 (Figure 5).

### 3.5. Keywords Analysis

**Figure 6:**
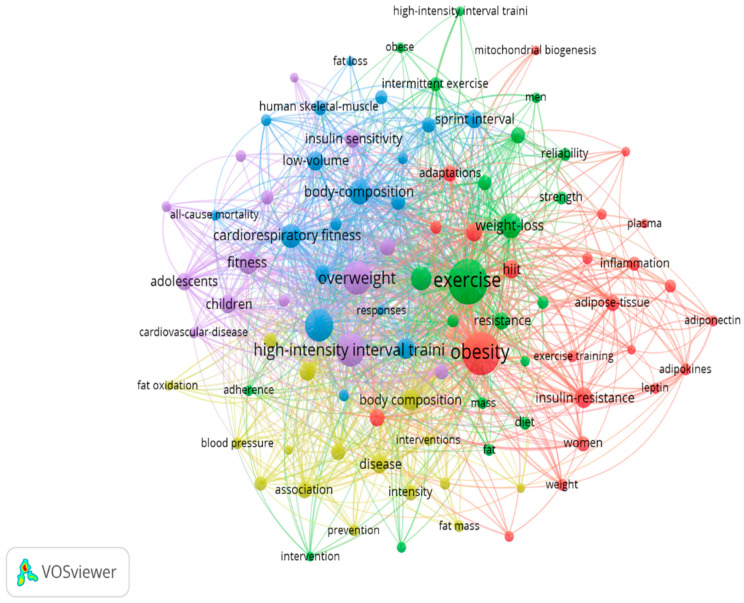
Keywords occurrence.

The most frequently used keywords are shown in Figure 6. For data extraction, a minimum of eight occurrences per keyword were established, with a total of 1120 terms found, but only 92 were included. The most frequently occurring words were “exercise” (*n* = 145), “obesity” (*n* = 131), “high-intensity interval training” (*n* = 81), “overweight” (*n* = 78), “physical activity” (*n* = 73), “body composition” (*n* = 46), “weight loss” (*n* = 45), “health” (*n* = 42), “cardiorespiratory fitness” (*n* = 40), and “fitness” (*n* = 38).

### 3.6. Citation and Country Analysis

**Figure 7:**
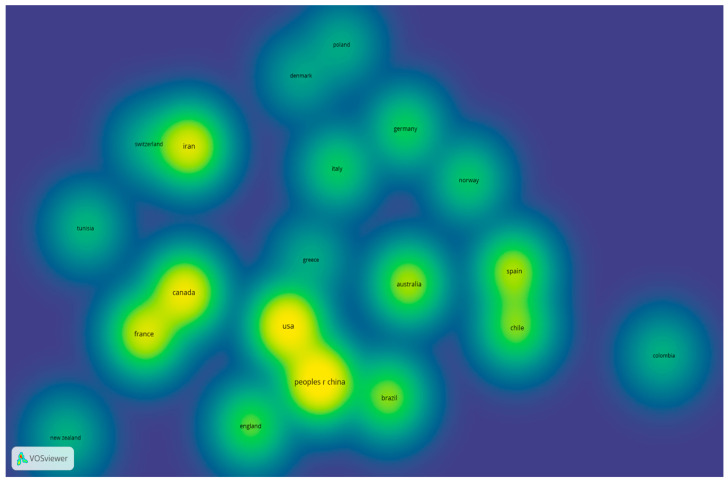
Citation by country.

Fifty-five country-based documents were found; however, 43 of them had at least two documents and five citations, as shown in Figure 7. The countries with the highest number of publications between 2011 and 2024 were the People’s Republic of China, with 888 citations and 49 publications; the USA, with 1447 citations and 44 publications; Canada, with 1200 citations and 37 publications; Iran, with 309 citations and 21 publications; and England, with 1083 citations and 18 publications. The only two South American countries reported were Brazil, with 466 citations and 21 publications, and Chile, with 346 citations and 20 publications.

### 3.7. Analysis of the Most Cited Studies

Table 6 presents the most cited review articles included in this study, showing the total number of citations received by each publication. This table identifies the works that have had the greatest academic impact within the field of study, reflected in their citation frequency. The articles listed address the application of HIIT in the context of obesity, as well as its effects on body composition variables, cardiorespiratory capacity, and metabolic markers. Likewise, the table allows us to observe the concentration of citations in a small number of publications, highlighting which reviews have been most influential in consolidating scientific knowledge on this topic.

Table 7 summarizes the most cited intervention articles. It reports the original studies that have received the highest number of citations, indicating their relevance and visibility within the scientific literature. The articles included mainly correspond to intervention trials that evaluate the effects of HIIT on anthropometric, physiological, and metabolic variables. The table shows the distribution of citations among different intervention studies, allowing us to identify those empirical works that have had the greatest impact and have contributed most significantly to the development and dissemination of knowledge in this field.

## 4. Discussion

The aim of this bibliometric review was to analyze the evolution and trends in the number of publications related to the study of HIIT in overweight and obese populations between 2011 and 2024. To this end, 282 documents indexed in the JCR were identified. The main findings reveal that (i) 2019 and 2022 were the years with the highest production; (ii) 2014 and 2019 are the years with the highest number of citations; (iii) the majority of documents are experimental studies with a large number of review studies; (iv) there are more studies on women, followed by children and, to a lesser extent, men and adolescents; (v) review studies have received more citations than intervention studies; (vi) the main journals publishing studies on HIIT and its relationship with obesity and overweight are as follows: Medicine and Science in Sport and Exercise, Sports Medicine, PloS One, Journal of Applied Physiology, Obesity Reviews, European Journal of Applied Physiology, Applied Physiology Nutrition and Metabolism; (vii) the most prolific authors are Aubertin-Leheudre, M. between 2021–2022, Kong, Z. in 2019, and Little, J. and Jung, M., who are the most prominent in 2018; (viii) the most prominent keywords are exercise, obesity, high-intensity interval training, overweight, physical activity, body composition, weight loss, health, and cardiorespiratory fitness; (ix) the country with the highest production is the United States.

The studies mentioned in Table 6 and Table 7 support the effectiveness of HIIT as an effective strategy for changing body composition and cardiovascular and metabolic variables, which are relevant considering that excess fat mass, loss of lean mass, and decreased cardiorespiratory fitness are independently correlated with an increased risk of morbidity and mortality [58,59,60].

For changes in body composition in relation to weight, according to the meta-analysis conducted by Jelleyman et al. [49], there was a significant reduction of 1.3 kg in body weight compared with that of the control group without exercise but not compared with that of the MICT group, an effect observed mainly in people described as overweight, obese, or at risk of type 2 diabetes. In terms of total abdominal, and visceral fat mass, HIIT significantly reduced abdominal (*p* = 0.007) and visceral (*p* = 0.018) fat mass. However, it is essential to establish that the best results are obtained when two strategies are combined: (i) PA and (ii) a low-calorie diet [51]. In this context, the study by Wewege et al. [50] compared the effects of HIIT and MICT on visceral fat using a reference instrument for noninvasive measurement based on computed tomography (CT) or magnetic resonance imaging (MRI). A significant decrease (19.5%) in abdominal visceral fat was detected after 12 weeks of HIIT on a treadmill, but no significant decrease was detected in the MICT group (11.1%). Notably, visceral fat deposits may have a greater impact on health than excessive total fat accumulation does [61], which is independently associated with health problems such as hypertension and insulin resistance [62,63].

For cardiovascular variables, García-Hermoso et al. [64] demonstrated in their study that HIIT programs generate greater reductions in systolic blood pressure, Standardized mean difference SMD = 0.39; equivalent to = 3.67 mmHg; *p* = 0.010) in overweight and obese young people than other forms of exercise do, although the mechanisms explaining these changes have not been fully elucidated. However, high-intensity PA can generate a greater reduction in sympathetic nervous activity [65,66] and an increase in nitric oxide-mediated vasodilation [67] than continuous moderate-intensity training. Similarly, García-Hermoso et al. [64] reported that HIIT has a greater positive influence on aerobic capacity than other forms of exercise do, particularly increasing VO2max in overweight and obese young people (SMD = 0.59; weighted mean difference of 1.92 mL/kg/min; *p* = 0.006). VO2max is an important marker of cardiometabolic health and is associated with a decreased risk of morbidity and mortality in the general population. Similarly, VO2max levels in youth predict cardiovascular disease later in life [68,69]. These results are consistent with a previous meta-analysis that reported statistically significant moderate effects in obese children for VO2max (SMD = 0.46) compared with a control group without exercise [70]. The authors establish that the improvement in VO2max through HIIT programs is due to an increase in oxygen availability from central effects (such as maximum cardiac output, total hemoglobin, and blood plasma volume) [71] and/or as a result of peripheral adaptations with an improved ability to extract and utilize available oxygen due to increased muscle oxidative potential [72,73]. Similarly, the effect of HIIT programs on skeletal muscle oxidative potential has been shown to be more pronounced than that of aerobic programs (moderate-intensity continuous training) [74,75].

Obesity is associated with an increase in metabolic disorders such as insulin resistance, type 2 diabetes, and cardiovascular disease [76]. Therefore, based on the variables analyzed in the studies, it is suggested that, compared with continuous exercise, HIIT is more effective at improving insulin resistance measures and that a control group without exercise should be included. Importantly, the greatest effects were observed in people with type 2 diabetes or metabolic syndrome. In addition, in people with type 2 diabetes or metabolic syndrome, there was a reduction of 0.92 mmol∙L^−1^ in fasting glucose and a reduction of 0.47% (5 mmol∙L^−1^) in HbA1c compared with studies with a control group without exercise [49]. The effect of exercise on improving peripheral insulin sensitivity, often associated with a reduction in body weight, has also been linked as one of the main mechanisms used to explain the increase in blood glucose after physical training and has been widely demonstrated after acute and chronic physical training [77]. There are several established metabolic pathways that are likely to be enhanced by HIIT, including skeletal muscle glucose uptake, muscle glucose transporter 4 (GLUT-4) content, and insulin sensitivity induced by muscle glycogen depletion [78].

The effects on the different variables mentioned depend on the characteristics of the intervention protocol in terms of the type of exercise. HIIT studies have compared running with cycling [51]. Run running promotes greater muscle mass recruitment for any given submaximal workload in relation to maximum capacity, which presumably leads to greater energy expenditure and a type of muscle contraction during running (concentric and eccentric) that contributes to greater fat oxidation at the same relative intensity. In addition, postexercise oxygen consumption in chronic obstructive pulmonary disease (COPD) patients is greater (+37%) after a running session than after a cycling session [50,79]. However, the suitability of running training for people with obesity needs to be clarified; therefore, the determination of the overall safety and sustainability of running protocols applied to overweight or obese individuals is limited.

High intensities (above 90% VO2max) appear to be more likely to reduce total body fat, whereas lower intensities are more effective at reducing abdominal and visceral fat mass. During high-intensity exercise (i.e., >65% VO2max), catecholamine responses increase significantly, promoting lipolysis through β-adrenergic receptors. The total abdominal area includes both subcutaneous and visceral adipose tissue. Since the content of β-adrenergic receptors is greater in visceral adipose tissue than in subcutaneous tissue, greater activation of the sympathetic nervous system (through the release of norepinephrine) during HIIT could explain why there is a greater dependence on visceral adipose tissue [80]. According to De Feo [81], the most efficient exercise program for obese individuals is to start at a moderate intensity and increase the intensity of the exercise by 5% every six training sessions until reaching 65% of maximum capacity.

Notably, even at low volumes, HIIT has been shown to be better than no exercise and superior to MICT, but it requires more time to improve cardiorespiratory fitness [82]. HIIT has been promoted as a time-saving form of exercise. Studies support that the health benefits of exercise can be achieved with just 21 ± 16 min of vigorous PA three times a week [49,50]. With respect to the duration of programs, even short-term HIIT (≤4 weeks) can improve VO2max compared with the control group in healthy populations, but moderate to long-term HIIT (>4–12 weeks) has shown additional beneficial effects compared with the control group and MICT in both healthy and overweight/obese populations [49].

Finally, in the study conducted by Wen et al. [83], a subgroup analysis was performed with different intervention protocols, which revealed that even short (≤30 s), low-volume (≤5 min), and short-duration (≤4 weeks) HIIT could have clear beneficial effects on VO2max compared with the control group. However, long intervals (≥2 min), high volumes (≥15 min), and moderate to long durations (≥4–12 weeks) of HIIT had significantly greater effects on VO2max than MICT or the control. Interestingly, when the HIIT group was compared with the control group and the HIIT group was compared with the MICT group, the effects of long-interval, high-volume HIIT were greater in healthy individuals, whereas long-term HIIT showed advantages in overweight/obese individuals. One of the important considerations of the authors is that short-interval HIIT produces training effects like those of sprint interval training (SIT) but involves a lower intensity with more repetitions. This means that although SIT is more time-efficient, short-interval HIIT could be an alternative approach when safety and feasibility issues regarding the application of HIIT in overweight populations are considered.

### 4.1. Implications for Public Health

This bibliometric review highlights the impact of PE on overweight and obese populations, especially HIIT. This highlights the need to identify the benefits of HIIT based on available scientific evidence to emphasize its use in different population groups. It is noteworthy that of the 282 documents included in this study, many documents do not specify the sample evaluated (65.6%), with low numbers of studies in children (6.4%) and young (1.4%). Likewise, it is reported that the study of HIIT in overweight and obese populations considers other health conditions of the population, although with low scientific output in diseases such as diabetes (3.2%) and cancer (1.1%). These findings express the need to develop a greater number of studies that consider determining the effects of HIIT on overweight and obese populations. Likewise, it would be necessary for public health systems to design PE plans under medical and professional supervision, seeking to reduce levels of physical inactivity [84] and promote public awareness of the different causes and complications derived from obesity in the different samples [85].

### 4.2. Limitations

This study represents the first bibliometric approach exclusively focused on the HIIT method in overweight populations, considering a broad period of analysis and multiple bibliometric indicators, exclusively with documents indexed in JCR. This research has several limitations that should be considered in future bibliometric studies analyzing HIIT. These are as follows: (i) heterogeneity in the population samples evaluated, as well as in the ages and characteristics of the participants; (ii) not limiting the search to certain databases, making it necessary to review multidisciplinary databases, such as Scopus, and others, such as ScienceDirect, SportDiscus, EMBASE, etc.; (iii) not considering secondary sources such as Google Scholar; (iv) the search period may limit recent advances or pioneering documents on the subject; (v) exclusive selection of documents indexed in JCR, knowing that other indexers exist, which may have left out several studies.

Prospects and practical applications.

It is necessary to review the number of weeks that HIIT interventions last to induce changes in the population samples evaluated. Importantly, each study details the main characteristics of the participants and variables evaluated with greater precision. For participants, it is necessary to recognize characteristic features such as age, sex, previous pathologies, level of overweight, level of physical condition, sports experience, habits, etc. Meanwhile, for interventions, it is necessary to know the weeks, days, and hours that the interventions last, intensities, volumes, exercises, sets, repetitions, etc. This will provide different protocols that help us to understand more clearly the effect of HIIT on overweight and obese populations. From a psychological perspective, it is important to understand how HIIT influences participants’ motivation and commitment and, from a neuroendocrine perspective, to analyze the hormonal response associated with different protocols and their relationship with fat reduction. Finally, more randomized controlled trials and clinical studies are needed to provide a clearer understanding of the effects of HIIT on the different morphological and physiological variables evaluated.

## 5. Conclusions

There has been an exponential increase in studies analyzing the effects of HIIT on excess weight individuals, with 2019 and 2022 being the most productive years. This has been accompanied by greater international academic cooperation led by countries such as the United States, China, Australia, Canada, and England through key concepts such as “physical activity”, “body composition”, “weight loss”, “health”, and “cardiorespiratory fitness”. There are also a greater number of documents evaluating women and, to a lesser extent, children, adult men, and young men. With respect to the population samples evaluated, the HIIT interventions focused only on overweight or obese individuals, and a low percentage of studies (6.4%) associated obesity with other diseases, such as diabetes, menopause, and cancer.

HIIT has been used as a means of inducing changes in body composition and physiological parameters in overweight and obese individuals. The main changes are associated with reductions in weight, body fat, body mass index, total cholesterol, and systolic blood pressure, as well as improvements in aerobic capacity measured through VO2max.

Finally, scientific research has contributed to understanding the relationship between HIIT and outcomes related to excess weight, with total fat reduction being one of the most consistently reported variables and no differences observed between sexes. HIIT could help reduce overweight and obesity in comparison with low-intensity training programs.

## Figures and Tables

**Table 1:** Overview of keyword searches, Boolean operators, and number of documents by type.

N° Search	Boolean Operators	Search Date	Publication Date Intervals	Total Number of Found Articles	Document Type	Number of Documents by Type
1	HIIT AND (overweight OR obesity)	28 September 2024	2011–2024	606–619	Article	516
					Review Article	80
					Early Access	11
					Meeting Abstract	10
					Proceeding Paper	1
					Retracted Publication	1
2	HIIT AND (overweight AND obesity)	29 September 2024	2011–2024	217–223	Article	173
					Review Article	43
					Early Access	3
					Meeting Abstract	3
					Proceeding Paper	1
3	(HIIT OR “High-Intensity Interval Training”) AND (overweight OR obesity)	30 September 2024	2011–2024	777–792 *	Article	617
					Review Article	104
					Meeting Abstract	41
					Early Access	12
					Letter	7
					Correction	4
					Editorial	2
					Proceeding Paper	2
					Book Review	1
					News Item	1
					Retracted Publication	1

Note: * This search was selected for the flow diagram because it had the highest number of records found.

**Table 2:** Types of published documents.

Document Type	Number of Documents	Percentage %
Research article	229	81%
Review	42	15%
Meeting summary	5	2%
Early access review	3	1%
Correction	1	0%
Editorial	1	0%
Letter	1	0%
Total	282	100%

**Table 3:** Types of published documents.

Document Type	Number of Documents	Percentage %
Articles (interventions)	232	82.3%
Systematic review and meta-analysis	16	5.7%
Meta-analysis	15	5.3%
Systematic review	6	2.1%
Randomized trial	5	1.8%
Systematic review and network meta-analysis	3	1.1%
Controlled trial	2	0.7%
Network meta-analysis	1	0.4%
Systematic review	1	0.4%
Umbrella review	1	0.4%
Total	282	100%

**Table 4:** Type of population.

Population	Number of Articles	Percentage %
Adults	67	23.80%
Females	58	20.60%
Males	37	13.10%
Not specified	31	11.00%
Adolescents	22	7.80%
Children	18	6.40%
Young Females	14	5.00%
Older adults	9	3.20%
Young adults	6	2.10%
Middle-age	6	2.10%
Young Males	5	1.80%
Male adolescents	4	1.40%
Female Adolescents	2	0.70%
Young	2	0.70%
Middle-aged males	1	0.40%
Total	282	100%

**Table 5:** Types of disease.

Disease/Condition	Number of Articles	Percentage %
Not applicable/Not specified	262	92.9%
Diabetes	9	3.2%
Postmenopause	6	2.1%
Cancer	3	1.1%
Aterosclerosis	1	0.4%
Mental health and polycystic ovary	1	0.4%
Total	282	100%

**Table 6:** Most cited papers on review articles.

Authors and Year of Publication	Participants Characteristics	Main Findings	Total Citation
Weston et al. [48]	Individuals with lifestyle-related chronic cardiometabolic diseases.	Improvements in the highest value reached during a test (VO_2peak_) with HIIT and MICT. HIIT showed greater VO_2peak_ increase. Both groups improved walking test performance and reduced systolic and diastolic blood pressure. HIIT reduced LDL and triglycerides, increased HDL, improved adiponectin, insulin sensitivity, beta-cell function, mitochondrial biogenesis, and Ca^2+^ reuptake. Greater nitric oxide and antioxidant availability. Improved left ventricular ejection fraction in heart failure patients.	824
Jelleyman et al. [49]	Participants with type 1 diabetes were excluded.	This review analyzed insulin resistance. HIIT reduced HOMA index compared to control group and continuous training group (CT). Insulin sensitivity improvement decreased over time after exercise session. Fasting glucose, with a reduction after HIIT, although not significant compared to control group. In individuals with metabolic syndrome or type 2 diabetes, reduction was greater. HbA1c showed no overall changes, but in individuals with metabolic syndrome/diabetes HIIT reduced HbA1c. Fasting insulin was reduced, but this reduction was not significant compared to control group. There were no significant differences compared to CT. VO2max increased after HIIT.	364
Wewege et al. [50]	It included apparently healthy individuals who were overweight or obese (mean age between 18 and 45 years). Overweight was defined as BMI > 25; obesity as a BMI > 30. Participants had no diagnoses of other medical comorbidities (e.g., coronary artery disease or diabetes).	This review showed that weekly training time was lower for HIIT than for MICT. Both HIIT and MICT significantly reduced body fat (1.7 kg and 2.1 kg, respectively) and waist circumference (3 cm each). There were no significant effects on body mass, lean mass, or trunk fat. In running protocols, both exercise methods had a greater impact on body fat reduction (2.6 kg) and a small effect on body mass. Cycle ergometer protocols showed no significant effects.	332
Maillard et al. [51]	Normal-weight and overweight/obese adults over the age of 18; high-performance athletes were not included.	La This review shows that HIIT reduces total fat mass, with running being more effective than cycling. High-intensity protocols are more effective than low-intensity protocols. Total fat reduction was greater in individuals with overweight/obesity, with no differences between sexes. HIIT reduces abdominal fat, with cycling being more effective than running. Low-intensity training was more effective than high-intensity in reducing abdominal fat, and the effect was significant only in individuals with overweight/obesity. For visceral fat, HIIT also shows reduction, especially in running. Low-intensity protocols were more effective in individuals with overweight/obesity, with no differences between sexes.	189
Petridou et al. [52]	Not applicable	Regarding HIIT, it is evident that it is advantageous because: it reduces body weight compared to groups that do not exercise, but is not more effective than moderate-intensity continuous training (MICT) in individuals with overweight or obesity. Both HIIT and MICT are equally effective in reducing body fat, although HIIT requires 40% less time. HIIT reduces body fat percentage more than traditional exercise. There are no differences in weight reduction, BMI, or waist circumference between both.	160

Note: HIIT: High-Intensity Interval Training; VO_2peak_: Peak Oxygen Consumption; HDL: High-Density Lipoprotein; MICT: Moderate-Intensity Continuous Training.

**Table 7:** Most cited papers on intervention articles.

Authors and Year of Publication	Participants Characteristics	Main Findings	Total Citation
Gillen et al. [53]	16 overweight/obese women. They were considered sedentary according to their usual self-reported BP (2 sessions/week of structured exercise of 30 min).	The authors, when experimenting with HIIT in overweight women, with and without prior fasting, observed that peak power and HRmax were associated with a subjective perception of effort with no differences between groups VO_2peak_ increased similarly in both groups, body mass had a reduction in abdominal, leg, and total fat percentage. HIIT also decreased abdominal and total fat mass, and lean mass tended to increase. Citrate synthase activity increased 23% without fasting and 22% in fasting, while β-HAD showed a greater trend in fasting. GLUT4 protein increased 42% without fasting and 61% in fasting.	162
Fisher et al. [54]	28 sedentary overweight/obese men (BMI 25–35 kg/m^2^; age 17–22 years). Criteria: BMI 25–35, sedentary lifestyle (<30 min/week of structured activity), normal glucose tolerance (fasting glucose < 100 mg/dL), no medications that affect metabolism, no smokers.	In this study, the HIIT group showed an average peak power of 810 watts in intervals and 140 watts in recovery, with heart rates of 178 BPM and 153 BPM, respectively. In the MIT group, average power was 138 watts and heart rate was 159 BPM. Both groups improved in several metabolic parameters: body fat reduction, total cholesterol, VLDL, HDL, triglycerides, insulin sensitivity index, QUICKI, and VO2peak. The MIT group improved VO_2peak_ more significantly than the HIIT group. ITT analysis showed a significant difference in diastolic blood pressure.	140
Martinez et al. [55]	20 adults (11 males, 9 females; age = 22 ± 4 years; BMI = 29 ± 3). Overweight or obese (BMI 25–35), insufficiently active (<3 days/week moderate BP), otherwise healthy.	This study shows that affect decreased over time in all trainings, with greater decrease in high-intensity continuous training and in 120 s intervals, compared to 30 s and 60 s intervals. There was a significant effect of training and time on enjoyment. The 30 and 60 s intervals were perceived as more pleasant than 120 s and continuous exercise. The 60 s training was perceived as more enjoyable than 120 s and continuous immediately after exercise. The trainings with 30 and 60 s intervals were perceived as more pleasant than 120 s and continuous exercise, especially after exercise.	139
Little et al. [56]	10 individuals were overweight/obese (BMI > 25 kg/m^2^; 8 women, 2 men; age = 40.6 ± 10.7 years). Inactive (<2 exercise sessions/week of ≥30 min). Data: height = 165.7 ± 6.2 cm, body mass = 99.6 ± 20.0 kg, BMI = 36.1 ± 7.1, fasting glucose = 5.6 ± 0.8 mmol/L.	In this study, HIIT generated a higher average heart rate and greater perception of effort compared to CMI. Both types of exercise increased perception of effort over time. Before lunch, glucose levels were higher in the HIIT group compared to CMI and the control group. HIIT and CMI significantly reduced the area under the curve of glucose after dinner compared to the control group. HIIT showed a greater reduction in postprandial glycemic response (PPS) to dinner. The day after exercise, the postprandial glycemic response to breakfast was also lower with HIIT and there was a reduction in PPS.	129
Vella et al. [57]	17 sedentary adults with overweight/obesity (7 males, 10 females; 18–44 years). Recruited from a university and surrounding community in the Pacific Northwest.	In this study, adherence to the exercise program was similar between MICT and HIIT, and both were equally enjoyed, with enjoyment scores between 74 and 125. Average daily energy expenditure was similar between groups. There was a greater decrease in LDL in the HIIT group compared to MICT after the intervention. There were no differences between groups in HDL cholesterol, triglycerides, total cholesterol, glucose, insulin, blood pressure, or waist circumference after 8 weeks. VO_2peak_ increased more in the HIIT group than in the MICT group. Postintervention IL-6 and CRP levels were different between groups, with greater changes in HIIT.	120

Note: HIIT: High-Intensity Interval Training; VO_2peak_: Peak Oxygen Consumption; HRmax: Maximum Heart Rate; β-HAD: Beta-Hydroxyacyl-CoA Dehydrogenase; GLUT4: Glucose Transporter Type 4; BPM: Beats Per Minute; MIT: Mitochondrial Integrity; VLDL: Very-Low-Density Lipoprotein; HDL: High-Density Lipoprotein; ITT: Insulin Tolerance Test; CMI: Cardiometabolic Index; MICT: Moderate-Intensity Continuous Training; IL-6: Interleukin-6; CRP: C-Reactive Protein.

## Data Availability

The data are not publicly available due to containing information that could compromise the privacy of research participants.
